# Multilevel factors affecting mental health literacy among older adults: a qualitative study based on social ecological model

**DOI:** 10.3389/fpubh.2025.1656116

**Published:** 2025-10-23

**Authors:** Yan Wang, Shoumei Jia, Anni Wang, Huiyuan Li, Rongjing Xu, Xuyan He

**Affiliations:** School of Nursing, Fudan University, Shanghai, China

**Keywords:** mental health literacy, aged, influencing factors, social ecological model, qualitative study

## Abstract

**Background:**

Mental health challenges among older adults are a growing global health priority. Mental health literacy (MHL) is a critical factor in mitigating these challenges and enhancing mental well-being. However, MHL levels among older adults remain relatively low, and its multilevel determinants are poorly characterized.

**Objectives:**

This study aimed to explore the multilevel factors influencing MHL among older adults from a dual perspective encompassing both older adults and geriatric healthcare providers, using a social ecological model (SEM).

**Methods:**

A qualitative descriptive study design was used. Semi-structured interviews were conducted with 15 older adults and 12 geriatric healthcare providers (doctors and nurses) in Shanghai between October 2024 and January 2025. Directed content analysis was guided by the SEM. The data were analyzed using NVivo 14.0.

**Results:**

Four SEM-aligned themes emerged as factors influencing MHL: (1) individual factors (digital health information acquisition capacity, psychological resilience, experiences with mental illness, and the perceived utility of engaging in mental health promotion activities and seeking professional assistance); (2) interpersonal factors (peer interactions and family functioning); (3) community factors (the intensity and modality diversity of mental health promotion activities, availability of mental health resources within healthcare institutions, and the sanitary conditions of residential environments); (4) societal factors (stigmatization of mental illness, privacy norms, as well as healthcare delivery systems and health insurance schemes).

**Conclusion:**

Using the SEM, this study explored the complex and multilevel factors that may influence older adults’ MHL. Older adults’ MHL is influenced by a combination of individual, interpersonal, community, and societal factors. Future studies should integrate culturally adaptive frameworks with policy-driven strategies to develop multilevel interventions encompassing interpersonal support systems, community resource coordination, and structural destigmatization programs for older populations.

## 1 Introduction

The global population is aging at an unprecedented pace, and older adults with mental health concerns are gaining prominence. According to World Health Organization (WHO) projections, the global population aged ≥60 years will reach 1.4 billion by 2030, meaning that approximately one in every six persons worldwide will be an older adult (1). The Chinese population aged ≥60 years has reached 260 million people, accounting for approximately 19% of the total population (2). As aging progresses, older adults commonly face multifaceted challenges, including declining physical function, cognitive changes, and social role transitions (3), demonstrating heightened mental health vulnerability relative to younger adult populations. Globally, the prevalence of mental disorders among older adults (≥60 years) is estimated at 14%. These disorders contribute 10.6% to the total years lived with disability (YLD) burden in this age cohort (4). China is experiencing a significant mental health crisis among its older population, with a reported prevalence of depressive at 20% (5) and anxiety disorders affecting 13% of older adults (6). Furthermore, the nation has the largest population of individuals with dementia globally (7). These mental health conditions significantly impair the quality of life, increase mortality risk, and exacerbate societal burdens, representing a major public health challenge.

Mental health literacy (MHL) is a crucial factor in improving individual mental health and preventing mental illness (8). The concept of MHL originated from “health literacy,” initially defined as “knowledge and beliefs about recognition, preventing, and managing mental disorders” (9). As research advances, the concept of MHL has continuously evolved and improved, gradually incorporating core elements, such as skills to support others affected by mental health concerns (10), mental health promotion (11), and help-seeking efficacies (12). In 2020, Chinese scholars Jiang et al. (13) introduced an integrated conceptualization of MHL from a culturally adapted perspective. They defined MHL as “the knowledge, attitudes, and behaviors that individuals acquire to promote their own mental health and that of others, as well as to manage their own and others’ mental illness” (13). This definition encompasses several key components: understanding knowledge related to mental health and mental illness, attitudes toward promoting one’s own and others’ mental health, attitudes toward coping with personal mental health concerns and providing support to others with mental illness, behaviors that promote one’s own and others’ mental health, and behaviors that address one’s own mental health concerns and support others with mental illness (13).

The literature (14) indicates that higher MHL significantly reduces the stigma associated with mental illness, facilitates help-seeking behaviors, and consequently enhances individual mental health. Conversely, low MHL constitutes the primary barrier to mental health service utilization (15). However, older adults globally exhibit substantially lower MHL than younger populations do. Age-related decline in physiological and cognitive capacities, limited mental health awareness (16), restricted access to health information, and diminished information-processing abilities (17) collectively exacerbate this disparity. A multisite review confirmed that older adults frequently misattribute mental illness symptoms to normal aging because of difficulties in accurate symptom identification (18). In China, the older adults’ MHL rate of 7.6% (19) is significantly lower than the national target of 30% by 2030. Therefore, identifying influencing factors is essential for developing targeted MHL interventions in this vulnerable population.

Current research on factors influencing MHL among older adults remains limited, with a predominant emphasis on sociodemographic variables (e.g., age, marital status, education, and socioeconomic status) (20). Although studies have demonstrated that social support (21) and mental health service accessibility (22) significantly predict MHL-related outcomes among younger populations, the mechanisms underlying these relationships in older adults remain poorly understood. Furthermore, few studies have explored the lived experiences of older adults regarding the determinants of MHL. Studies relying exclusively on older adults’ perspectives have inherent limitations in that the capacity of older adults to perceive macro-level MHL determinants (e.g., policy resources and community services) may be constrained by their health status, cognitive decline, and restricted mobility (23). Conversely, geriatric healthcare providers, as frontline service implementers, possess experiential knowledge of both geriatric engagement and policy-resource dynamics, offering critical insights into MHL determinants (24). Thus, a complementary dual-perspective qualitative approach integrating older adults and geriatric healthcare providers is essential to comprehensively understand the determinants of MHL among older population.

The social ecological model (SEM) emphasizes the interrelated interactions between individuals and their surrounding environments, encompassing the individual level, interpersonal level, organizational level, community level, and societal level (25). It is widely applied in fields such as mental health and public health to explore influencing factors or develop interventions (26). For instance, Zhu et al. (27) used SEM to identify factors that influence organizational health literacy in meeting the needs of geriatric populations, highlighting the combined impact of multiple factors on the organizational health literacy of healthcare institutions. Therefore, the SEM offers a systematic and thorough theoretical framework for exploring the determinants of older adults’ MHL. It transcends narrow individual-level concerns to systematically examine the broader factors influencing MHL among the older population. Notably, within retirement context, diminished engagement with organizations reduces the relevance of organizational-level determinants for older adults’ MHL. Concurrently, health service organizations are integral components of community-level systems for this population.

Hence, grounded in Jiang’s culturally adapted definition of MHL, this study employed SEM to guide the interview and analytical structure for the results. The investigation focused on the multilevel factors (individual, interpersonal, community, societal) influencing MHL among older adults through semi-structured interviews with both older adults and geriatric healthcare providers.

## 2 Methods

### 2.1 Design

This study was guided by a post-positivist paradigm, which emphasizes the use of systematic and rigorous methods to approximate objective reality while acknowledging the inherent influence of researcher subjectivity and contextual limitations (28). A descriptive qualitative approach was employed to investigate the views and feelings of older adults and geriatric healthcare providers regarding factors influencing MHL among older adults. The methodology prioritizes participants’ language in natural settings to represent their views and feelings with minimal interpretation (29). The study was conducted in compliance with the Consolidated Criteria for Reporting Qualitative Research (COREQ) (30) (Supplementary material 1).

### 2.2 Research team

The research team consisted of two professors, one young researcher, and three graduate students specializing in geriatric mental health and community care. The team members had extensive experience in qualitative research and published numerous related papers. The first author (YW) was a female graduate student with training in qualitative research methods and had participated in qualitative research projects (31).

### 2.3 Participants, recruitment, and setting

Participants were recruited using purposeful sampling methodologies. For transferability, participants were recruited based on the principle of maximum diversity, considering older adults’ differences in sex, age, education, and previous mental illness diagnoses. Geriatric healthcare providers were recruited based on their institutional tier, years of work experience, and professional roles. Between October 2024 and January 2025, 15 older adults and 12 geriatric healthcare providers were recruited from the Putuo and Minhang districts in Shanghai. Putuo District is one of the urban districts in Shanghai with the largest older population, with approximately 380,000 people aged ≥60 years in 2023, whereas the suburban Minhang District had approximately 410,000 people aged ≥60 years. Eligible older adults willing to participate were purposively sampled from community health centers (CHCs) in both districts, with recruitment sites encompassing outpatient clinics, rehabilitation facilities, and community activity centers.

The inclusion criteria for older adults were as follows: age ≥60 years, no hearing or language impairments, clear consciousness and ability to communicate normally, and voluntary participation. The exclusion criteria were as follows: concomitant severe physical illness, severe mental illness rendering the individual unable to respond, neurocognitive dysfunction, severe disability, bedridden status, and other conditions rendering the individual unable to respond. The inclusion criteria for geriatric healthcare providers were as follows: those working in geriatric healthcare, have been working in their current position for ≥3 years (24), and willing to participate. Individuals with communication barriers or clinically assessed impaired comprehension capacity were excluded. The sample size was determined through information saturation. When interview data exhibited repetitive content and no new subcategories or categories emerged, this indicated that information saturation had been reached. In this study, after interviews began to yield redundant information, two additional older adults and two geriatric healthcare providers were interviewed. Upon confirming that no new subcategories or categories emerged, the interviews were concluded. Ultimately, the data saturation was confirmed after 27 interviews. Participants were identified by coded numbers, with “P” and “N” representing older adults and geriatric healthcare providers, respectively.

### 2.4 Data collection and analysis

The preliminary interview outline was developed based on the content of MHL using the SEM framework informed by a comprehensive literature review and research team deliberations. Subsequently, pilot interviews were conducted with an older adult and a geriatric healthcare provider to ascertain the clarity, comprehensibility, and precision of the questions. Pre-interviews were not included in the final analysis. Informed by the feedback received from these pre-interviews, certain technical terms were rendered more colloquial, culminating in the final version (Supplementary material 2). To ensure comfort and minimize disruption, the participants recruited face-to-face interview locations, including community chronic disease education offices, private homes, and healthcare providers’ offices. Prior to conducting the interviews, the research objectives and content were explained to the participants, who voluntarily signed informed consent forms. During the interviews, with the participants’ consent, on-site audio recordings were made. Concurrently, notes were taken to document the participants’ gestures and facial expressions, thereby enhancing the accuracy of data collection. Before concluding the interview, the researcher offered a succinct review and synthesis of the information gathered during the discussion, subsequently soliciting the interviewee’s perspective on any potential additional insights. All interviews were conducted privately by the first author, with only the interviewee and the researcher present, and had an average duration of 31 min. No repeat interviews were carried out, nor did any participant withdrawal occur. Verbatim transcription was performed within 24 h, followed by accuracy verification by another researcher (RX). Verified transcripts were returned to the participants for member validation prior to analysis. The final data were managed using NVivo 14.0.

Data collection and analyses were performed simultaneously. This study employed the directed content analysis method recommended by Hsieh and Shannon (32) to analyze the data. The analytical framework was established prior and structured around the four levels of the SEM—individual, interpersonal, community, and societal—which served as the core categories, each with predefined subcategories. The analysis commenced with an immersive reading of the interview transcripts to ensure deep familiarity with the data. Meaningful units were then systematically identified and coded. This was followed by a comparative analysis and critical review of the generated codes, which were subsequently grouped into the predefined subcategories. This process was iterative, facilitating the refinement of the subcategories. For example, the broad subcategory of “capability” at the individual level was specified as “digital health information acquisition capacity,” whereas “resource allocation” at the community level was operationalized as “availability of mental health resources within healthcare institutions” to more accurately represent the real data. To avoid potential analytical limitations associated with predefined categorical frameworks, all semantic units that could not be classified under the initial categories were systematically documented. The research team subsequently engaged in a collective discussion to evaluate these units as critical issues, determining their alignment with existing categorical frameworks or the need for the development of new categories. Ultimately, all data were integrated into a refined SEM framework. This process was performed independently by two researchers (YW, RX), with consensus reached through discussion. A third researcher (SJ) was introduced to review and arbitrate findings.

### 2.5 Rigor

No prior relationships existed between the researchers and the participants. To reinforce the rigor of the research, the team undertook systematic training in qualitative methodologies prior to data collection and analysis. The interviewer engaged in mock interviews to cultivate the ability to “bracket” preconceptions, thereby ensuring that the interviews genuinely reflected participant perspectives. During the analysis phase, two researchers independently applied a unified analytical framework. All analytical judgments were traced back to the raw data and refined through discussion to reach consensus, ensuring that the findings were firmly grounded in the data. A third researcher reviewed and arbitrated the findings. For instance, the iterative refinement of subcategories was exemplified by the evolution of the community level subcategories for “mental health promotion activities.” Initially, researchers YW and RX independently generated the distinct codes “intensity of publicity” and “diversity of outreach formats” from the data. Through comparative analysis and discussion, it was determined that these codes represented two critical, complementary facets of a single predefined subcategory. This consensus, reviewed by a third researcher (SJ), led to the operationalization of the subcategory as “the intensity and modality diversity of mental health promotion activities,” thereby enhancing its precision and fidelity to the empirical evidence. Furthermore, data collection and analysis were conducted concurrently. The constant comparison of preliminary findings with newly gathered data facilitated the identification of connections and discrepancies, thereby enhancing the study’s credibility and overall trustworthiness. Purposeful maximum diversity sampling was employed to select participants based on a range of characteristics (sex, age, education, institutional tier, etc.), ensuring the transferability of the findings. Moreover, the findings of this study were presented with quotes from the participants. All interview transcripts and associated materials were systematically backed up and managed using NVivo 14.0 software to enhance the auditability of the study.

### 2.6 Ethical considerations

This study adhered to the ethical principles of the Declaration of Helsinki and was approved by the Ethics Committee of the School of Nursing at Fudan University (IRB#2024-04-2). All the participants received geriatric-adapted consent disclosures detailing anonymity safeguards and data encryption protocols. Written informed consent was obtained after confirming the participants’ comprehension.

## 3 Results

### 3.1 Participants’ characteristics

The sample consisted of 27 participants: 15 older adults and 12 geriatric healthcare clinicians. The older adult participants was predominantly female, with ages ranging from 60 to 83 years, and most had junior or senior high school education. Five older adults had previously been diagnosed with a mental illness. The geriatric healthcare clinicians represented tertiary, secondary, and primary care institutions and rural clinics, with 50% of them having more than 10 years of clinical experience. The clinical roles included nursing (*n* = 6) and doctors (*n* = 6). Comprehensive demographic characteristics are presented in Tables 1, 2.

**Table 1:** Characteristics of older adults (*n* = 15).

Participants	Sex	Age	Education	Previous mental illness diagnoses	Place of residence
P1	Female	60	Primary school	None	Minhang district
P2	Male	83	College	None	Putuo district
P3	Female	72	College	None	Putuo district
P4	Female	68	Senior high school	None	Putuo district
P5	Male	70	Primary school	None	Minhang district
P6	Male	70	College	None	Putuo district
P7	Male	74	College	None	Putuo district
P8	Female	60	Primary school	Anxiety	Minhang district
P9	Female	68	Junior high school	Anxiety	Minhang district
P10	Female	69	Junior high school	None	Putuo district
P11	Male	69	Junior high school	None	Minhang district
P12	Female	70	Senior high school	Depression	Putuo district
P13	Female	65	Senior high school	None	Minhang district
P14	Female	80	College	Anxiety	Putuo district
P15	Male	70	Senior high school	Anxiety	Minhang district

**Table 2:** Characteristics of geriatric healthcare providers (*n* = 12).

Participants	Institutions	Years of working experience	Geriatric professional roles
N1	CHCs	26	Nurse
N2	CHCs	5	Doctor
N3	CHCs	20	Doctor
N4	Psychiatric specialty centers	4	Nurse
N5	Tertiary comprehensive hospitals	25	Doctor
N6	Rural clinics	6	Doctor
N7	Rural clinics	4	Doctor
N8	Tertiary comprehensive hospitals	23	Nurse
N9	Tertiary comprehensive hospitals	27	Nurse
N10	CHCs	5	Doctor
N11	Psychiatric specialty centers	10	Nurse
N12	Psychiatric specialty centers	11	Nurse

### 3.2 Factors influencing MHL among older adults

**Figure 1:**
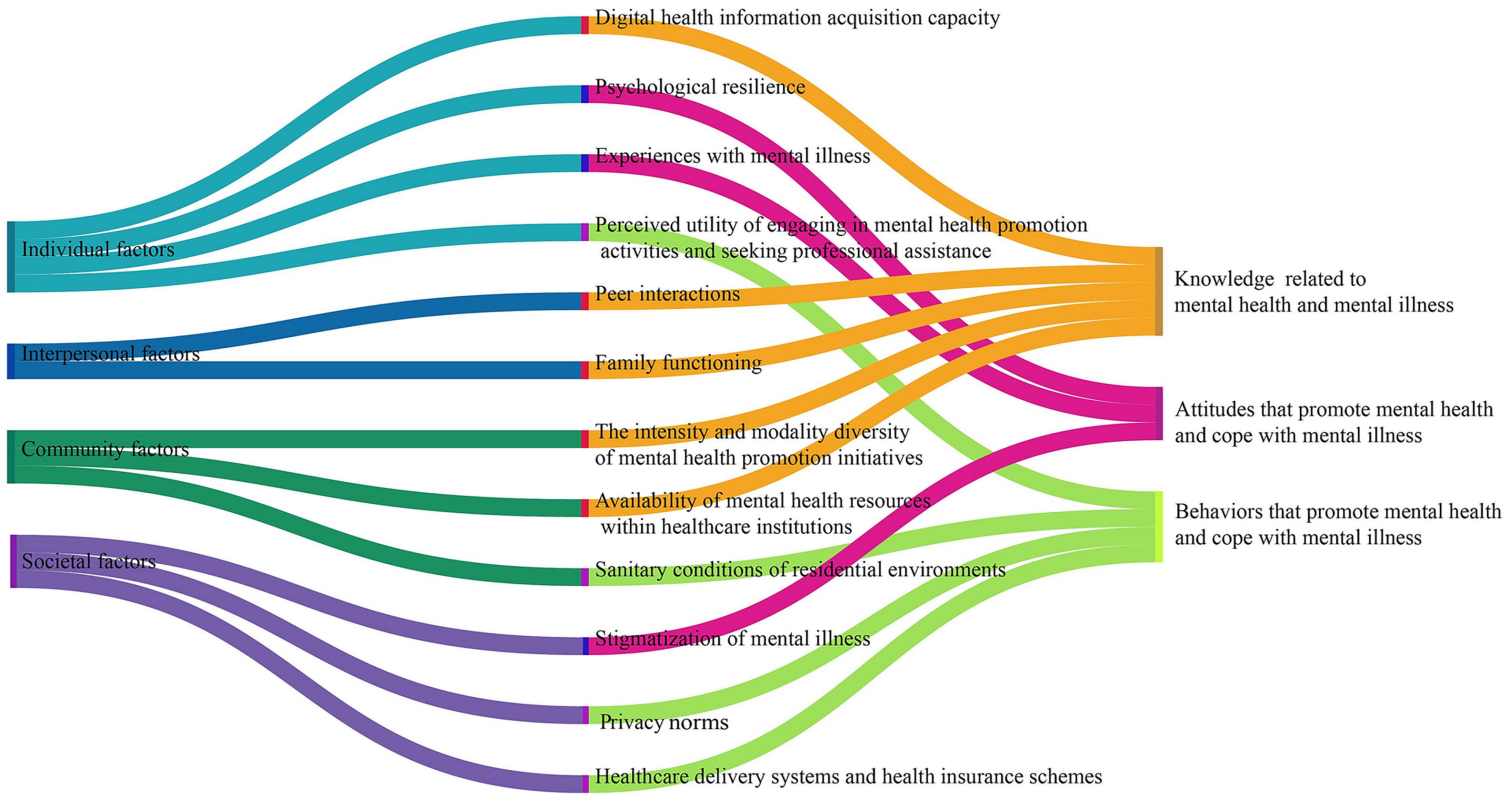
Factors influencing MHL among older adults.

Guided by SEM, 12 factors influencing MHL (knowledge, attitudes, and behaviors) were extracted across four categories (Figure 1).

#### 3.2.1 Category 1: individual factors

Individual factors refer to the physiological and psychological characteristics, social attributes, and behavioral competencies of individuals. This study examined digital health information acquisition capacity, experiences with mental illness, psychological resilience, and the perceived utility of engaging in mental health promotion activities and seeking professional assistance.

##### 3.2.1.1 Digital health information acquisition capacity

Older adults who were capable of accessing information through digital devices reported that obtaining mental health information via mobile platforms enhanced their understanding of the clinical manifestations of mental illness (e.g., depression), associated risks, and mental health promotion strategies.

“With today’s advanced internet accessibility, I frequently use ‘WeChat’ (a dominant Chinese social media application) to read public accounts. I’ve learned that persistent unhappiness, appetite loss, and insomnia may indicate depression. This condition can become severe, potentially leading to suicidal ideation, which is profoundly concerning.” (P10).

“I regularly access mental health content through my smartphone, including instructional yoga videos. Incorporating these mobile-guided yoga sessions has provided perceived benefits for my psychological well-being.” (P14).

Conversely, some participants believed that older adults lacking proficiency in utilizing digital devices for internet access might be constrained in their understanding of mental health and mental illnesses.

“Based on my observations, limited smartphone-mediated internet engagement among older adults constrained the knowledge of mental health, including recognition of psychiatric disorders.” (N7).

“I do not understand things like anxiety or depression. Mainly because I cannot use the mobile phone, so my daughter usually helps me with calling and such tasks.” (P2).

##### 3.2.1.2 Psychological resilience

Psychological resilience refers to an individual’s capacity to maintain stability or recover from adversity (33). Some participants believed that older adults with high psychological resilience tend to rely on self-regulation and generally have a low willingness to seek help from external sources to cope with mental health concerns.

“I feel my current mindset is quite ‘beautiful’; I can self-regulate, so even with such issues, I see no need to seek others’ help.” (P6).

“I believe my psychological endurance is quite strong, and I can self-regulate, making external help unnecessary.” (P4).

“Among the older adults I’ve encountered, the majority of them perceive their psychological resilience and recovery capacity as quite good, believing they can self-regulate their moods. Therefore, when coping their own mental illness, they usually adopt a more passive attitude and are unwilling to seek help from others.” (N9).

##### 3.2.1.3 Experiences with mental illness

Personal experience with mental illness shapes older adults’ attitudes toward others’ mental illness. Older adults who had a personal history of mental illness demonstrated stronger empathy and willingness to support their peers who had mental health concerns.

“They still need help—it’s truly distressing. Having struggled with anxiety myself, which once confined me indoors, I consistently encourage those with mental health concerns.” (P14).

“I regularly invite friends who struggled with mental illness to practice Eight Brocades with me. Having experienced depression in 2006, I deeply understand their emotional state.” (P12).

Conversely, older adult participants who had no lived exposure to mental illness tended toward social avoidance of others.

“Mainly because I’ve never experienced such problems. I know people with depression tend to act extremely, so I’m not very willing to interact with them.” (P5).

“I’m unwilling because I’ve never had such problems.” (P10).

##### 3.2.1.4 Perceived utility of engaging in mental health promotion activities and seeking professional assistance

Perceived benefits drive older adults’ engagement in health-promoting behaviors. Most participants believed that older adults perceive minimal benefits from mental health promotion activities, and generally refrain from participating in related activities.

“Initially, I attended mental health lectures and similar activities, but later only participated in community health checkups. Because I felt their other mental health activities were not very helpful to me.” (P3).

“From my mother’s perspective, she does not routinely engage in such activities (mental health promotion activities). She often perceives these lectures as yielding few benefits, and rarely takes initiative to learn about related topics.” (N8).

Concurrently, some healthcare providers reported that when older adults perceive limited benefits from professional support, they generally do not proactively seek such assistance.

“Many older adults I encounter believe mental health consultations may not necessarily be effective, with minimal perceived benefits. They often feel we clinicians are profiting from them, leading many to adopt an attitude of discouraged resignation and avoid hospital visits.” (N8).

#### 3.2.2 Category 2: interpersonal factors

The interpersonal level encompasses an individual’s closest social relationships and immediate support networks, including family members, intimate partners, friends, and colleagues. Interpersonal factors manifest as external influences, such as familial support or neglect, and peer modeling or pressure. In this study, peer interactions and family functioning were identified.

##### 3.2.2.1 Peer interactions

Peer interactions serve as a significant source of mental health and mental illness knowledge among older adults. Most older adult participants reported that sharing their mental healthcare experiences with peers improved their comprehension of mental health promotion and the underlying factors influencing mental illness.

“I often chat with classmates in our WeChat group. You see, there is one member who often shares music and tells us that listening to music can improve mood. There are also people who often advise maintaining positive outlooks, increasing outdoor activities, and exploring new environments, emphasizing emotional well-being as integral to health.” (P2).

“Casual conversations with friends yield valuable insights. For instance, I used to habitually stay up late, but my badminton partner mentioned casually that sleep deprivation could worsen mood. That’s when I realized: ‘Oh! Staying up late actually affects emotional state!’” (P7).

##### 3.2.2.2 Family functioning

Family functioning refers to emotional bonding, communication, intimacy, and adaptability to external events among family members (34). Older adults in families with weak emotional bonds (e.g., those with geographically distant children or those living alone) experience inadequate intergenerational support and dwindling access to mental health promotion and mental illness prevention knowledge.

“Ah (looking down), my son is abroad. He’s extremely busy with work and seldom calls us, scarcely communicating about mental health concerns. My understanding in this area is limited.” (P4).

“Like my child working far away, they rarely visit and scarcely communicate about mental wellness topics. I consider myself fortunate if they do not cause me trouble.” (P15).

“Older adults whose children seldom visit them or who live alone lack family communication channels. Nobody explains how to maintain positive moods or depression risk factors, resulting in limited understanding of these topics.” (N6).

#### 3.2.3 Category 3: community factors

Community level generally refers to the broader living environment and social networks that encompass an individual. It transcends single organizational boundaries and includes larger collectives, such as geographical communities and virtual communities, along with their interaction patterns. Community-level factors include infrastructure, resources, public safety, and environmental security. This study identified the intensity and modality diversity of mental health promotion activities, availability of mental health resources within healthcare institutions, and the sanitary conditions of residential environments.

##### 3.2.3.1 Intensity and modality diversity of mental health promotion activities

Most participants reported that the intensity and modality diversity of mental health promotion activities in the current community exhibit generally low participation rates among older adults, thus constraining their understanding of mental health promotion.

“Regarding promotional efforts, we have observed predominantly lecture-based formats with limited variation. Unfortunately, these fail to sufficiently engage older adults and consequently may restrict their understanding of mental health concerns.” (N4).

“My neighborhood conducts annual gynecological and colorectal screenings, but provides minimal mental health promotion. My knowledge in this domain limited.” (P13).

##### 3.2.3.2 Availability of mental health resources within healthcare institutions

Hierarchical disparities in healthcare resources have resulted in inequitable access to mental health services for older adults. Most geriatric healthcare providers from secondary and tertiary medical institutions reported that their institutions had relatively abundant mental health resources, enabling the timely provision of professional mental health services that enhance older adults’ knowledge of mental health and mental illness.

“Our institution has psychiatric nurses, certified psychological therapists, and psychiatrists who conduct flexible educational sessions for older adults and can help enhance older adults’ understanding of conditions like depression.” (N12).

“Our psychiatry department holds biweekly mental health education for patients and family caregivers. We cover precipitating factors of common conditions (e.g., depression) and introduce group art therapy approaches. This educational initiative increases older adults’ comprehension of these disorders.” (N9).

However, four primary care providers and several older adults reported deficient mental health resources in their local CHCs, which constrained older adults’ knowledge of mental health promotion, mental illness symptoms, and treatment options.

“Given our village’s limited resources, lacking professional counselors or mental health initiatives, older residents know little about improving their mental state.” (N7).

“We lack specialized mental health clinics here. Establishing a dedicated unit could heighten older adults’ mental health awareness, potentially increasing their understanding of symptoms and treatments for conditions like depression or bipolar disorder.” (N1).

“I know little about mental illness. Our community hospital has no psychiatric department, and I do not know the symptoms and treatment approaches for depression, anxiety, etc. …” (P11).

##### 3.2.3.3 Sanitary conditions of residential environments

Some participants regarded clean and orderly living environments as behavioral enablers that enhance older adults’ willingness to engage in outdoor activities, facilitate social participation, and foster healthy behavioral patterns.

“My neighborhood environment is relatively favorable, unlike disorderly neighborhood, so my spouse and I frequently walk our dog downstairs. I find this benefits our psychological wellbeing.” (P3).

“The comprehensive sanitary improvements in our village positively impact older adults. They report greater happiness, and more seniors willingly join neighborhood-organized psychological health activities.” (N6).

“Environmental factors contribute significantly. Many residential areas now feature well-maintained living environments. When surroundings are pleasant, older adults become more inclined to exercise or socialize downstairs, facilitating healthy habit formation.” (N10).

#### 3.2.4 Category 4: societal factors

Societal factors encompass the macro-environmental elements across society, including sociocultural values, economic and social policies, legislation, and media discourse. This study identified the stigmatization of mental illness, privacy norms, as well as healthcare delivery systems and health insurance schemes as societal factors.

##### 3.2.4.1 Stigmatization of mental illness

Some participants reported stigma surrounding mental illness in Chinese society. Older adults generally have a low willingness to seek help from external sources to cope with their own mental illness.

“People still treat mental illnesses as somewhat taboo, they view depression negatively. So, I generally avoid discussing it with others (family or friends) or asking for help.” (P7).

“Given our cultural traditions, discrimination persists against conditions like depression. Many consider it ‘being crazy’, a negative label. Most older adults I encounter deny having such conditions when mentioned and resist consulting mental health professionals or seeking support.” (N2).

##### 3.2.4.2 Privacy norms

Several participants reported that the emphasis on privacy within Chinese culture norms may attenuate the willingness to intervene in others’ mental health concerns.

“I cannot do much either, primarily because it involves another person’s privacy. People nowadays place significant importance on privacy, making it inappropriate for outsiders to intervene.” (P5).

“Given the current high value placed on personal privacy, there is little I can do to assist individuals with mental health condition.” (P3).

“I do not want to meddle in others’ affairs; respecting their privacy means I should not pry.” (P7).

“Privacy is highly valued today. Older adults in this community tend to focus on their own matters and rarely interfere in the affairs of others.” (N8).

##### 3.2.4.3 Healthcare delivery systems and health insurance schemes

Primary geriatric healthcare providers reported insufficient specialized mental health training for primary care physicians, coupled with inadequate implementation of streamlined referral mechanisms. Consequently, older adults who require psychological assistance frequently encounter delays in obtaining accurate information. This systemic service gap often prevents them from seeking professional help to cope with their mental health concerns.

“Despite the current policy emphasis on primary care physicians, we face significant challenges. Older patients present with mental health concerns, yet primary care physicians often lack the specialized training needed to provide accurate guidance in this domain. Furthermore, the referral process remains cumbersome. Although policies stipulate priority access for referrals, this is not effectively operationalized in practice. Many older patients have expressed frustration to me regarding these barriers, perceiving healthcare access as overly burdensome and consequently avoiding hospital visits for such concerns.” (N3).

Compounding these access barriers, several participants highlighted that many older adults in rural areas depend primarily on the Rural Cooperative Medical Scheme (RCMS) for healthcare reimbursement. The significantly lower reimbursement rates offered by the RCMS compared with that offered by urban health insurance programs frequently deter them from actively pursuing professional assistance for psychological issues.

“Being from a rural area myself, I receive less reimbursement for hospital visits than my urban counterparts. Consequently, I typically avoid seeking hospital care for minor ailments or when experiencing emotional distress.” (P1).

“In my experience, many older adults exhibit symptoms such as anxiety. However, since the majority of rural older adults are enrolled in the RCMS, which provides lower reimbursement rates for hospital services than urban insurance, they rarely seek specialist mental health services for these conditions.” (N7).

## 4 Discussion

Guided by the SEM, this study transcends an individual-centric approach by offering a systematic framework for the development of interview guides and the analysis of the multilevel factors that influence older adults’ MHL (knowledge, attitudes, and behaviors). The incorporation of dual perspectives—from both older adults and geriatric healthcare providers—and the use of triangulation strengthened the validation of the multilevel factors influencing MHL among older adults. The findings affirm the explanatory value of the SEM’s four-tiered structure in MHL among older adults, revealing that MHL is not an isolated capacity but is embedded within a multilayered environmental context. Specifically, the findings reveal that factors at the individual (digital health information acquisition capacity, psychological resilience, experiences with mental illness, perceived utility of engaging in mental health promotion activities and seeking professional assistance), interpersonal (peer interactions, family functioning), community (the intensity and modality diversity of mental health promotion activities, availability of mental health resources within healthcare institutions, sanitary conditions of residential environments), and societal levels (stigmatization of mental illness, privacy norms, healthcare delivery systems and health insurance schemes) collectively shape and drive the development of MHL among older adults. These findings offer a comprehensive understanding of the factors influencing MHL among older adults and provide a reference for designing integrated MHL promotion strategies.

Among the individual factors, we extracted four factors, and digital health information acquisition capacity was considered a key factor critically shaping mental health and mental illness knowledge. Although mobile devices were highlighted as key health information conduits, digital health information acquisition capacity deficits were found to impede access, particularly for those with limited technological proficiency. This aligns with established evidence demonstrating that digital interventions can increase individuals’ understanding of mental illness, improve their willingness to seek help, and reduce the stigma associated with mental illness (35). Previous studies have shown that digital technologies facilitate the dissemination of health information (36) and promote social interaction, thereby alleviating social isolation (37). Thus, it is imperative to develop targeted interventions focused on enhancing digital skills among older adults. We recommend that relevant personnel design age-appropriate digital skills training programs. Such programs could incorporate, for instance, large-print illustrated manuals, dialect-narrated commentaries, and slow-motion instructional videos. This approach may optimize older adults’ access to information pertaining to mental health and psychiatric conditions. Paradoxically, psychological resilience exhibited dual-directional effects on MHL. Although resilience typically correlates positively with MHL (38), our data revealed that older adults exhibiting higher levels of psychological resilience demonstrate a reduced willingness to seek external support. Analysis suggests that this phenomenon may be attributable to individuals with elevated resilience potentially overestimating their self-efficacy, which could lead to an underappreciation of the severity of mental health issues and a corresponding decrease in motivation to pursue assistance (39). Therefore, future interventions should preserve resilience while calibrating an accurate risk perception among older adults, particularly regarding clinical thresholds for professional intervention. Furthermore, attitudes toward others with mental illness were significantly determined by older adults’ mental illness experiences. Those with mental illness experience demonstrated greater empathy and supportiveness, aligning with evidence that experiential exposure reshapes attitudinal frameworks (40, 41). Finally, the perceived utility of engaging in mental health promotion activities and seeking professional assistance was identified as a pivotal driver of health behaviors in older adults. Low perceived utility was pervasive in our study, reducing participation and help-seeking initiations. This finding aligns with Elshaikh et al. (42), who reported that negative perceptions of mental health services were a primary deterrent to seeking professional help among older adults. This resonates with Perceived Value Theory (43), wherein positive utility appraisals potentiate behavioral intentions. Thus, enhancing the perceived value of mental health engagement may also function as a strategy to promote the mental health and well-being of older adults.

Among the interpersonal factors, we extracted two that highlighted the centrality of social support ecosystems. Peer interactions served as the predominant conduit for mental health knowledge transfer among the older adult respondents. This aligns with the evidence that 39% of older adults acquire mental health knowledge via informal networks (40). This suggests that structured peer support groups offer a scalable mechanism for disseminating MHL. At the same time, family functions are also considered to influence older adults’ mental health and knowledge of mental illness. This resonates with evidence linking suboptimal family functioning to constrained communication, reduced information access, and attenuated mental health awareness (44). Consequently, it is imperative that relevant personnel focus intensely on older adults living alone or with inadequate family support, facilitate communication and emotional exchange between older adults and their family members, thereby encouraging the active participation of older adults in social activities and fully leveraging the protective role of healthy family dynamics, further promoting MHL among older adults.

At the community level, three factors were identified that revealed the centrality of mental health services and environmental enablers. The availability of mental health resources within healthcare institutions and the intensity and modality diversity of mental health promotion activities are recognized as key factors influencing older adults’ mental well-being and knowledge of mental illness. In this study, CHCs geriatric healthcare providers universally reported critical shortages of mental health specialists and fragmented service delivery. This aligns with Adusei’s et al. (45) findings. As frontline healthcare gatekeepers (46), primary health institutions (PHIs) optimize service accessibility through geographical proximity (47). This underscores the need for the relevant authorities to strengthen the mental health capacity of PHIs through specialist deployment and dedicated service units. Evidence confirms that diverse mental health outreach modalities enhance older adults’ engagement and MHL (24). Current community mental health promotion activities are characterized by infrequent implementation and format rigidity (predominantly didactic), which reduce older adult engagement. These findings suggest that community practitioners should implement multimodal strategies that incorporate interest-based activities, micro-videos, and community radio. Additionally, the scaled implementation frequency and expanded reach amplify knowledge diffusion. Notably, we revealed a previously undocumented pathway: sanitary conditions of the residential environment modulate health-promoting behavioral habits. Participants reported that improved environmental sanitation increased older adults’ motivation for outdoor activities and social participation, thereby catalyzing healthier behavioral patterns. This aligns with *Chinese Citizens’ Health Literacy: Basic Knowledge and Skills (2024)* linking environmental optimization to reduced psychological distress and enhanced community engagement (48). Consistent with this evidence, age-friendly environmental design (encompassing sanitation, housing, and transport) facilitates social participation (49). Thus, synergistic improvements in community hygiene and age-friendly infrastructure represent actionable strategies to promote mental health behaviors and cultivate MHL-sustaining habits.

Among the societal factors, we extracted three that profoundly reflect how the macroenvironment shapes individual behaviors and attitudes. The stigmatization of mental illness impedes older adults’ willingness to seek help for mental health concerns. This echoes the WHO’s emphasis on the strong stigma surrounding mental health concerns, which is a key barrier to seeking help (4). This is consistent with Hyseni Duraku’s et al. (50) findings. Traditional Chinese philosophies (Confucianism, Taoism, and Buddhism) portray mental illness as personal failure (51), which constitutes a key contributing factor. Motivated by a desire to preserve face (social dignity and reputation), many older adults are reluctant to seek external help for fear of being labeled a “failure” (52). Notably, despite China’s collectivist orientation, a subset of older participants exhibited a privacy preservation orientation regarding others’ mental illness, perceiving such conditions as private matters and typically refraining from intervention. This is consistent with Liu et al.’s findings (53). Two interrelated factors may explain this phenomenon: (1) increasing societal emphasis on personal privacy protection to prevent potential conflicts amid social development and (2) traditional Chinese values prioritizing family integrity, where health-related matters are considered inappropriate for public disclosure (54). These findings indicate that anti-stigma interventions should extend beyond public education to normalize mental health discourse and dismantle stereotypes (55). Community support initiatives should simultaneously respect cultural sensitivities and develop mutual assistance models that are compatible with local privacy norms. Finally, healthcare delivery systems and health insurance schemes significantly shape help-seeking behaviors among older adults. Although China has significantly strengthened its primary care infrastructure through enhanced service capacity and expanded primary care physicians training (56), structural barriers persist. Geriatric healthcare providers who participated in the study described three key barriers: (1) limited mental health competencies in primary care institutions, (2) inefficient referral coordination, and (3) inadequate reimbursement under rural health insurance schemes. Collectively, these factors impede access to professional help, particularly for older adults in rural areas. This finding is similar to the findings of Shahraki-Mohammadi et al. (24), highlighting that inefficiencies in mental health service delivery arise from the lack of a standardized policy framework, while insufficient social welfare funding emerges as a critical determinant of low mental health service uptake. This outcome also parallels the findings of Abdelmonaem et al. (57), who identified the substantial financial burden of healthcare costs as a primary factor influencing students’ decisions to seek professional psychological assistance. Consequently, during the implementation of healthcare reforms, it is imperative for relevant personnel to enhance the mental health service capacity of primary healthcare providers. This enhancement can be accomplished through initiatives such as regular skills training. Concurrently, research suggests that healthcare providers may exhibit a tendency to neglect the implementation of referral procedures due to a lack of incentives (58). Therefore, relevant personnel should contemplate the establishment of a structured clinical supervision mechanism and an incentive scheme to optimize referral efficiency, thereby ensuring the safety and rights of patients. Lastly, systematically increasing reimbursement rates for older adults healthcare services in rural areas may effectively encourage the utilization of mental health services among older adults.

## 5 Limitations of this study

This study employed the SEM to explore the multilevel determinants of MHL among older adults through semi-structured interviews with both older adults and geriatric healthcare providers. However, the study had several limitations that warrant further investigation. Although methodological rigor was strengthened through dual independent coding with consensus discussions and third-party review and arbitration of discrepancies, the interpretive nature of qualitative research inherently precludes the complete elimination of analytical subjectivity. Despite maximum variation sampling, the geographical restriction to Shanghai districts limits generalizability and necessitates cautious extrapolation. Therefore, caution should be exercised when extrapolating these findings beyond the cultural context of this study.

## 6 Implications for policy and practice

In light of the findings of this study, we recommend a strategy that integrates digital skills training with mental health education to enhance the capacity of older adults to independently access mental health information and manage their mental health. Concurrently, practitioners should cultivate support networks among the relatives and friends of older adults, alongside implementing regular mental health promotion activities, to facilitate the natural incorporation of mental healthcare into the daily lives of older individuals. Efforts to develop age-friendly communities should systematically incorporate mental health components into community planning, thereby enhancing the visibility and accessibility of mental healthcare support within everyday environments. At the healthcare system level, mental health services should be extended to primary care institutions through their integration into essential public health services. Primary care institutions should establish dedicated mental health units, while competencies in mental health assessment and brief intervention should be recognized as core professional criteria for primary care providers, thereby enhancing the quality of grassroots mental health service delivery. Furthermore, ongoing healthcare reforms should prioritize the strengthening of mental health support in rural areas. Expanding insurance coverage and increasing reimbursement rates for mental healthcare services would alleviate financial barriers for rural older adults and contribute to narrowing the urban–rural gap in mental health services.

## 7 Conclusion

This study explored the multilevel factors influencing MHL among older adults through semi-structured interviews with older adults and geriatric healthcare providers based on the SEM. The findings reveal that MHL among older adults is collectively shaped by determinants spanning individual, interpersonal, community, and societal levels. This study advances our current understanding of geriatric MHL determinants and provides insights for practitioners. Future interventions should adopt a systemic perspective that comprehensively addresses and integrates these multilevel determinants to achieve sustainable improvements in MHL among older adults.

## Data Availability

The raw data supporting the conclusions of this article will be made available by the authors, without undue reservation.
