# Supplementary material for: Multilevel factors affecting mental health literacy among older adults: a qualitative study based on social ecological model
Source: Front Public Health. 2025 Oct 23;13:1656116. doi: 10.3389/fpubh.2025.1656116 (PMC12589026; doi:10.3389/fpubh.2025.1656116)
Supplement: Supplementary file 2 [file Table_2.DOCX]

Supplementary Material 2

Interview Guide

| Participants | Question |
| --- | --- |
| Older Adults | 1. Knowledge related to mental health   - What strategies or approaches for promoting mental health are you aware of? - What are the sources from which you learned this information?   2. Knowledge related to mental illness   - What do you think are the most common mental illness among older adults? - What factors influence how well you understand these mental health conditions? (From individual, interpersonal, community, and societal perspectives)   3. Attitudes toward the promotion of one's own and others' mental health   - What is your perspective on promoting mental health for oneself and other older adults? - Why do you hold this view? (From individual, interpersonal, community, and societal perspectives)   4. Attitudes regarding coping with personal mental health concerns   - If you were personally experiencing symptoms of a mental health concern (e.g., persistent sadness, anxiety, confusion), how would you perceive that situation? - What factors do you believe would shape your perception of your own mental health concerns? (From individual, interpersonal, community, and societal perspectives)   5. Attitudes regarding providing support to others with mental illness   - How do you generally perceive older adults who live with diagnosed mental illness? - What factors influence these perceptions? (From individual, interpersonal, community, and societal perspectives)   6. Behaviors that promote one's own and others' mental health   - What actions do you typically take to enhance your own mental well-being and support the mental well-being of other older adults around you? - What factors influence your behaviors to engage in these activities promoting mental health for yourself and for others? (From individual, interpersonal, community, and societal perspectives)   7. Behaviors for addressing one's own mental health concerns   - If you were experiencing symptoms of a mental health concern, what would you do? - What factors would you consider in making your decision? (From individual, interpersonal, community, and societal perspectives)   8. Behaviors for supporting others who have mental illnesses   - What can you do for older adults around you who have mental illness ? - What factors influence your decision? (From individual, interpersonal, community, and societal perspectives) |
| Geriatric healthcare providers |  |
|  | 1. Knowledge related to mental health and mental illness   - What do you understand about mental health and common mental illnesses among older adults? - What factors do you think influence one’s understanding of mental health and mental illness among older adults? (From individual, interpersonal, community, and societal perspectives)   2. Attitudes toward the promotion of one’s own and others’ mental health   - Based on your interactions with older adults, how do older adults view the promotion of their own mental health and that of others? - What factors do you think shape their attitudes (as described above) toward mental health promotion? (From individual, interpersonal, community, and societal perspectives)   3. Attitudes toward coping with personal mental illness and providing support to others with mental illness   - What are the attitudes you have observed among older adults toward their own mental illness and that of others? - What do you think are contributing factors that underlie the formation of older adults’ attitudes described above (toward their own mental illness and that of others)? (From individual, interpersonal, community, and societal perspectives)   4. Behaviors that promote one's own and others' mental health   - What are the behaviors you have observed among older adults engaging in promoting their own mental health and that of others? - What factors do you think influence older adults in engaging in behaviors that promote their own mental health and that of others? (From individual, interpersonal, community, and societal perspectives)   5. Behaviors that address one’s own mental illness and support others who have mental illness   - What do you think are the behaviors that older adults commonly engage in to cope with their own mental illness or that of others?   What factors influence the behaviors of older adults in coping with mental illness (either personally or in others)? (From individual, interpersonal, community, and societal perspectives) |
